# Sustained, local delivery of the PARP inhibitor talazoparib prevents the development of mammary gland hyperplasia in *Brca1*-deficient mice

**DOI:** 10.1038/s41598-020-79663-7

**Published:** 2021-01-13

**Authors:** Di Zhang, Bijay Singh, Jessica Moerland, Owen Mitchell, Lizbeth Lockwood, Sarah Carapellucci, Srinivas Sridhar, Karen T. Liby

**Affiliations:** 1 Department of Pharmacology and Toxicology, Michigan State University, B430 Life Science Building, 1355 Bogue Street, East Lansing, MI 48824 USA; 2 Theranano LLC, 41 Esty Farm Road, Newton, MA 02459 USA; 3 Northeastern University, Boston, MA USA

**Keywords:** Cancer, Drug discovery

## Abstract

Mutations in *BRCA* genes are the leading cause of hereditary breast cancer. Current options to prevent cancer in these high-risk patients, such as anti-estrogen drugs and radical mastectomy, are limited by lack of efficacy, undesirable toxicities, or physical and emotional challenges. We have previously shown that PARP inhibitors can significantly delay tumor development in BRCA1-deficient mice. Here, we fabricated the PARP inhibitor talazoparib (TLZ) into spacer implants (InCeT-TLZ) for localized and sustained delivery. We hypothesized that this novel formulation will provide an effective chemopreventive strategy with minimal toxicity. TLZ was released gradually over 30 days as implants degraded. InCeT-TLZ significantly decreased proliferation and increased DNA damage in the mammary glands of BRCA1-deficient mice. Notably, the number of mice that developed hyperplasia in the mammary glands was significantly lower with InCeT-TLZ treatment compared to the control group. Meanwhile, InCeT-TLZ was also better tolerated than oral TLZ, without loss of body weight or anemia. This study provides proof of concept for a novel and safe chemopreventive strategy using localized delivery of a PARP inhibitor for high-risk individuals. Future studies will directly evaluate the effects of InCeT-TLZ for preventing tumor development.

## Introduction

Breast cancer is the most commonly diagnosed cancer and the second leading cause of cancer deaths in women in the United States. In 2019, 268,600 women were expected to be diagnosed with breast cancer in the U.S. The breast cancer-associated (*BRCA1*) gene is an important tumor suppressor that protects against genomic instability^1^. *BRCA1* regulates numerous cellular functions, including cell cycle, apoptosis and transcription^2,3^. Moreover, the BRCA1 protein plays a critical role in repairing double-stranded DNA breaks through the homologous recombination (HR) pathway. Mutations in *BRCA* genes are the leading cause of hereditary breast cancer. Women with *BRCA* mutations have up to an 80% lifetime risk of developing breast cancer^4^. The majority of *BRCA1* mutated tumors are basal-like^5^, which is a subtype associated with a poor prognosis^6^. Developing effective cancer prevention strategies has become essential for these BRCA carriers.

Cancer prevention aims to lower the risk of developing cancer^7^. Effective cancer prevention strategies not only diminish the physical problems and emotional distress caused by cancer but also reduce the financial burden of healthcare. Lifestyle modifications are important primary prevention strategies for cancer. Accumulating evidence has suggested that better dietary choices^8^, eliminating the use of tobacco^9^, maintaining physical activity and proper body weight^10^ can reduce cancer risk.

Besides lifestyle changes, there are also medical interventions available for cancer prevention. The selective estrogen receptor modulators (SERMs) are successful examples of drugs that can prevent breast cancer. Tamoxifen and raloxifene have been approved by the FDA to prevent breast cancer in high-risk women^11^. However, they have limited or no effects on preventing BRCA-deficient breast cancer^12,13^. The undesired side effects and increased risk of endometrial cancers further hinder BRCA carriers from taking these agents^14^. Radical mastectomy is another option to prevent breast cancer in these high-risk patients. Prophylactic mastectomy reduces the risk of breast cancer by at least 89% in *BRCA1* and *BRCA2* carriers^15^. Nonetheless, radical mastectomy brings a huge burden to patients both physically and emotionally. Other options are desperately needed for patients who do not accept radical mastectomy.

PARP inhibitors were developed to treat BRCA-deficient cancers by inducing synthetic lethality^16,17^. Both BRCA and PARP (poly (ADP-ribose) polymerase) are essential for proper DNA repair. BRCA initiates DNA repair of double-strand DNA breaks though the homologous recombination repair pathway, while PARP mediates DNA repair of single-strand DNA damage. Inhibiting PARP by PARP inhibitors in BRCA-deficient cells results in genomic instability and induces cell death. Olaparib and talazoparib are two PARP inhibitors that have been approved by the FDA for the treatment of BRCA-mutated breast cancer^18^. Talazoparib is approximately 100 times more potent than olaparib because in addition to inhibiting the catalytic activity of PARP, talazoparib traps PARP at the site of DNA damage, i.e. “PARP trapping,” which further induces cell death^19^.

In addition to their efficacy for treating breast cancer, we have previously reported that PARP inhibitors are also effective for preventing the development of mammary gland tumors in BRCA1-deficient mice. When fed in diet, veliparib or olaparib (200 mg/kg diet or approximately 50 mg/kg body weight) delayed the average age of the first detectable tumor by 2.4 weeks and 6.5 weeks, respectively, compared to controls^20^. Currently, PARP inhibitors are formulated for oral delivery. Low bioavailability with oral delivery of these drugs, poor drug accumulation in the target tissue and undesired toxicities because of high systemic exposure limit the use of these drugs for prevention.

In the present studies, we aim to improve local delivery of PARP inhibitors for development as effective chemopreventive agents with minimal toxicities. The PARP inhibitor talazoparib was fabricated into spacer implants (InCeT-TLZ), which can be injected directly into mammary glands using brachytherapy needles. This formulation using biodegradable polymers was designed to induce a sustained release of talazoparib for localized therapy. Poly (lactic-co-glycolic) acid (PLGA) has been approved by the FDA as a biodegradable and biocompatible copolymer for drug delivery. PLGA has flexible mechanical and degradation properties that have been widely used for developing controlled delivery systems for many different molecules^21^. Here, InCeT-TLZ was first tested in vitro to validate its physicochemical properties, including stability, loading efficiency, release kinetics and biological functions. InCeT-TLZ was then tested in vivo using *Brca*^*Co/Co*^*;MMTV-Cre;p53*^+/−^ mice to evaluate its efficacy and toxicity profiles. To determine if InCeT-TLZ is effective for prevention, we first tested if these inserts can alter biomarkers before palpable tumors can be detected.

## Materials and methods

### Fabrication of implant

All solvents used were HPLC grade and purchased from Sigma-Aldrich unless otherwise stated. Talazoparib (SelleckChem, TX), poly(lactic-co-glycolic) acid (PLGA, MW 7–17 kDa) (Sigma-Aldrich, MO), 1 mL tuberculin syringes (Becton, Dickinson, NJ) and SPX-50 silicone tubing (Saint-Gobain, MI) were purchased for implant fabrication.

Talazoparib implants were fabricated using a polymer extrusion method with modifications^22^. The implants were prepared with a composition of 5% (w/w) talazoparib embedded in poly(lactic-co-glycolic) acid matrix. Accordingly, talazoparib (9 mg) was dissolved in dimethyl sulfoxide, and PLGA (190 mg) was separately dissolved in chloroform. The polymer and drug solutions were mixed thoroughly by vortexing and sonication. The polymer/drug mixture was transferred to a 1 mL syringe and extruded into silicone tubing using an infusion pump (Harvard Apparatus) at a rate of 2.5 µL/min. After extrusion, the tubing was kept in an oven at 60°C overnight. The next day, the implants formed inside the tubing were ejected, cut to an appropriate size and stored in a closed vial at − 20°C. Blank PLGA implants without drug (empty spacers) were fabricated in the same method described above.

### Characterization of implant

A Hitachi S-4800 (Tokyo, Japan) field emission scanning electron microscope (SEM) was used to observe cross-sections of the implants. To prepare the samples for SEM analysis, the implants were dipped in liquid nitrogen to flash freeze and a cross-section of the cylindrical implant was cut with a frozen razor. The cut implants were mounted on SEM stubs using conductive carbon adhesive and sputter-coated with 10–15 nm of platinum for 5 min using a Denton Vacuum DV-502 system (Moorestown, NJ). The implants were imaged using SEM at 5–15 kV.

### Drug loading and releasing studies

The drug loading and releasing behavior of InCeT-TLZ implants was determined by high-performance liquid chromatography (HPLC) using an Agilent 1260 Infinity II system and a reverse phase SUPELCOSIL LC-18 HPLC column. The samples were run by HPLC using a mobile phase of acetonitrile and water (1:1), both solvents containing 0.1% H_3_PO_4_, with a gradient flow rate of 0.8 mL/min, and detected with ultraviolet absorbance at 230 nm. To determine the drug loading per unit length (mm) of implant, 4 mm long implants (n = 3) were first dissolved in dimethylformamide (0.1 mL), ethanol (0.4 mL) was added, and the extracted drug was quantified by HPLC analysis. A standard curve was fit with a linear regression curve and the curve was used to quantify the concentration of talazoparib in each implant. To determine the drug releasing behavior of the implants, 4 mm long TLZ implants (n = 3) were incubated with 1 mL of PBS (pH 6.0) in a microcentrifuge tube at 37 °C. At scheduled time intervals, the release medium was entirely withdrawn from the microcentrifuge tube and replaced with 1 mL of fresh PBS. The amount of talazoparib released in the withdrawn medium was quantified by HPLC using a standard curve. The drug released from the implants was plotted as a function of time.

### Cell culture

W0069 and W780 cells were derived from mammary tumors of BRCA1-deficient mice and provided by Dr. Chuxia Deng at the NIH (Bethesda, MD)^23^. Cells were cultured in DMEM media + 10% FBS + 1% Pen/Strep (Corning Cellgro, Mediatech, Manassas, VA). W0069 and W780 cells were treated with empty spacer or InCeT-TLZ (2 mm or 4 mm) in 6-well plates for 48 h, and then cells were harvested and proteins were extracted for western blotting. In the cell viability assay, cells were seeded into 96 well plates and treated with empty spacer or InCeT-TLZ (1 mm) for 5 days. Cell viability was assessed by the MTT assay.

### Western blotting

Western blotting was performed as previously described^24^. W780 and W0069 cells treated with empty spacer or InCeT-TLZ were lysed in RIPA buffer (1 M Tris–Cl, 5 M NaCl, pH 7.4, 0.5 M EDTA, 25 mM deoxycholic acid, 1% triton-X, 0.1% SDS) with protease inhibitors (1 mM PMSF, 2 µg/mL aprotinin and 5 µg/mL leupeptin). The BCA assay was used to quantify the concentration of protein samples. 20 µg of protein were loaded and separated by 10% SDS-PAGE gels and transferred to nitrocellulose membranes. Primary antibodies were applied to detect the corresponding proteins, including γH_2_AX (Abcam, 1:1000), cleaved-caspase 3 (c-caspase 3, Cell Signaling Technology, 1:1000), PARP/cleaved-PARP (Cell Signaling Technology, 1:1000), PCNA (Santa Cruz, 1:1000), and vinculin (Cell Signaling Technology, 1:4000). Secondary antibodies (anti-rabbit or anti-mouse linked to HRP) were purchased from Cell Signaling Technology. ECL Western blotting substrate (GE Healthcare Life Sciences, UK) was used to detect the signal. Images shown are representative of 3 independent experiments.

### In vivo studies

**Figure 1:**
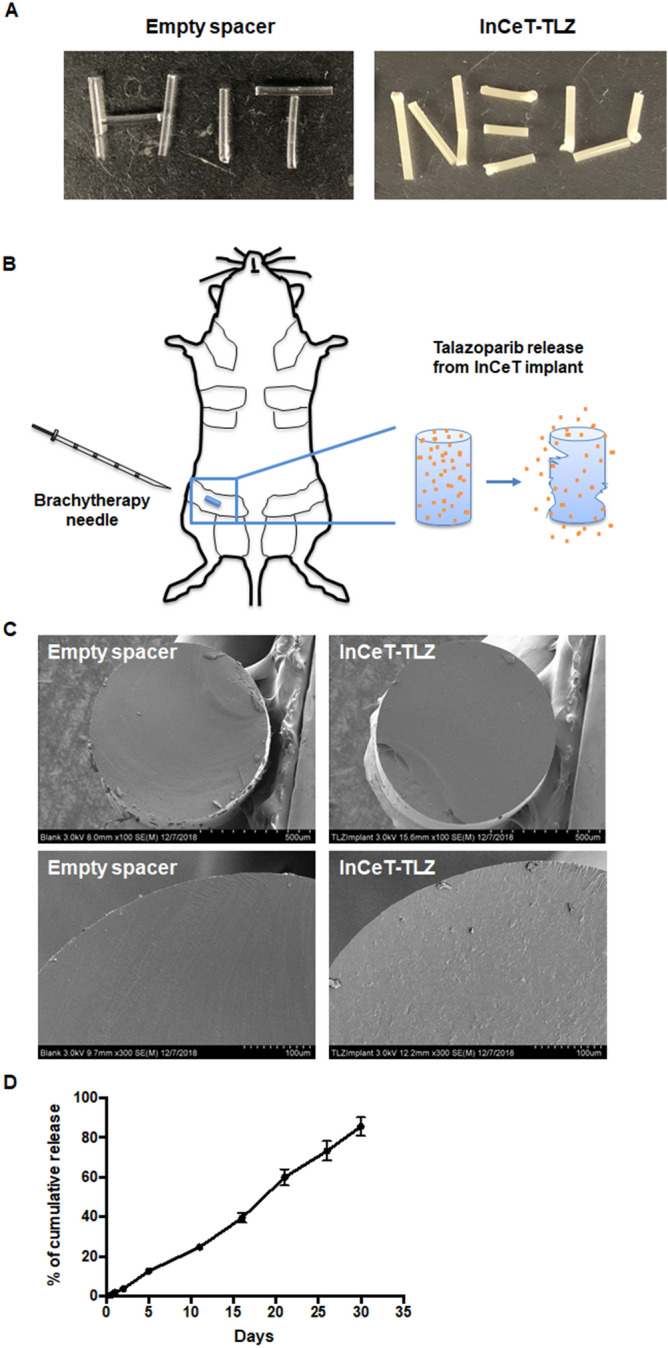
Characterization of the InCeT-TLZ implant. (**A**) Pictures of PLGA spacers with (right) or without (left) talazoparib. (**B**) Diagram of the implantation of InCeT-TLZ. InCeT-TLZ can be inserted into a mouse mammary gland using a 18G needle to puncture the skin and create a path for a brachytherapy needle. As PLGA degrades, drug is released locally into the mammary gland. (**C**) Scanning electron microscope (SEM) images of flash-frozen and sectioned implants. (**D**) The release kinetics of talazoparib from the implants in PBS medium (pH 6.0) at 37 °C. N = 3 implants. Data shown as mean ± SE.

All protocols were carried out ethically in accordance to the Regulations for the Management of Laboratory Animals at Michigan State University. All experimental protocols for the ethical use of animal studies were approved by the Institutional Animal Care and Use Committee at Michigan State University (protocol 201800050). Every effort was made to minimize suffering. Mice were euthanized by inhalation of carbon dioxide followed by cervical dislocation. Age-matched, and when possible littermate-matched, female BRCA1-deficient mice (*Brca*^*Co/Co*^*;MMTV-Cre;p53*^+/−^) were randomized into three groups (N = 33–35/group): InCeT-TLZ group (2 mm in length with a total drug load of 50 μg), empty spacer group (2 mm of blank PLGA InCeT implant), and oral TLZ treatment group (FreeTLZ, 50 μg TLZ was divided into 13 injections and given 3 times a week over 4 weeks, which is 3.85 μg/injection and approximately 0.13 mg/kg body weight). All implants were inserted into the left abdominal mammary gland (Fig. 1). Three different cohorts with different starting times for treatment (12, 16, or 20 weeks of age) were included. Weights of the mice were monitored twice a week. All the mice were harvested after 4 weeks of treatment. Complete blood counts were performed using an Idexx Procyte Dx hematology analyzer when the mice were harvested. Mammary glands were collected for biomarker analysis.

### Immunohistochemistry

Immunohistochemistry was conducted as described previously^22^. Brca1^Co/Co^;MMTV-Cre;p53^+/−^ mice were treated with empty spacer, InCeT-TLZ or FreeTLZ for 4 weeks. Mammary glands (left abdominal, which received the implant) were then harvested and sectioned for histopathology and immunohistochemistry. Citrate buffer (Vector, Cat. # H3300) was used for antigen retrieval. Slides were incubated in 3% hydrogen peroxide for 10 min to quench the endogenous peroxidase activity. Sections were stained with PCNA (1:200, Santa Cruz) or γH_2_AX (1:100, Abcam) antibodies for 1 h at room temperature or overnight at 4°C, respectively. Anti-mouse and anti-rabbit secondary antibodies conjugated to HRP were purchased from Cell Signaling. Signal was detected using a DAB kit (Cell Signaling Technology). Sections were counterstained with hematoxylin (Vector). The percentage of positively stained cells was quantified using ImageJ. All the analysis was done blinded as to group identity to eliminate any potential bias.

### Statistical analysis

The in vitro experiments were repeated at least three times. Results were expressed as mean ± SE. For the in vivo experiments, results were analyzed using one-way ANOVA followed by a Tukey test if the data fit a normal distribution; the Kruskal–Wallis one-way ANOVA on ranks was used followed by the Dunn test for multiple comparisons if the data did not fit a normal distribution (Prism 6)^24^. A paired t-test was used to compare body weight before and after treatment. For the histopathology of mammary glands, a Chi-Square test was used to compare proportions^24^. p < 0.05 was considered statistically significant.

## Results

### Fabrication and characterization of InCeT-TLZ implant

InCeT-TLZ implants were fabricated using a solvent-based polymer extrusion method. The fabrication procedure is simple, reproducible and cost-effective. The implants produced are solid cylindrical rods (Fig. 1A), which are stable at room temperature. These spacer implants can be injected to the mouse mammary gland or human breast tissue directly using brachytherapy needles. As PLGA degrades, talazoparib will be released slowly in situ (Fig. 1B). SEM images of flash-frozen and sectioned implants showed a smooth, homogeneously compacted surface without pores (Fig. 1C). After loading the drug, the InCeT-TLZ implant showed a slightly rough surface because of the intercalated drugs (Fig. 1C).

To determine the consistency of drug loading in the implants, the amount of drugs in the implants produced in two small-scale batches were compared by HPLC. The loading content of TLZ in two different batches was approximately 25 µg per unit length (mm) of implant. Moreover, HPLC chromatography confirmed the physical stability of the drug in the implants. HPLC data showed the retention time of a peak of talazoparib in the implant had the same peak as the standard talazoparib in the chromatogram.

The release profile of talazoparib from the implants was also studied. To determine the release kinetics, InCeT-TLZ implants were cut to predetermined lengths and incubated in PBS medium (pH 6.0) at 37 °C. At each predetermined time point, the medium was completely removed and replaced with fresh medium. Each collected medium fraction was subjected to HPLC analysis to quantify the released drug. The release profile of talazoparib implants showed continuous drug release in vitro (Fig. 1D). There was no burst release of drug within the period of 30 days. The release of drug from the implant was 85.66 ± 4.65% of the loading content by 30 days.

### InCeT-TLZ induces DNA damage and cell death in BRCA1-deficient tumor cells

**Figure 2:**
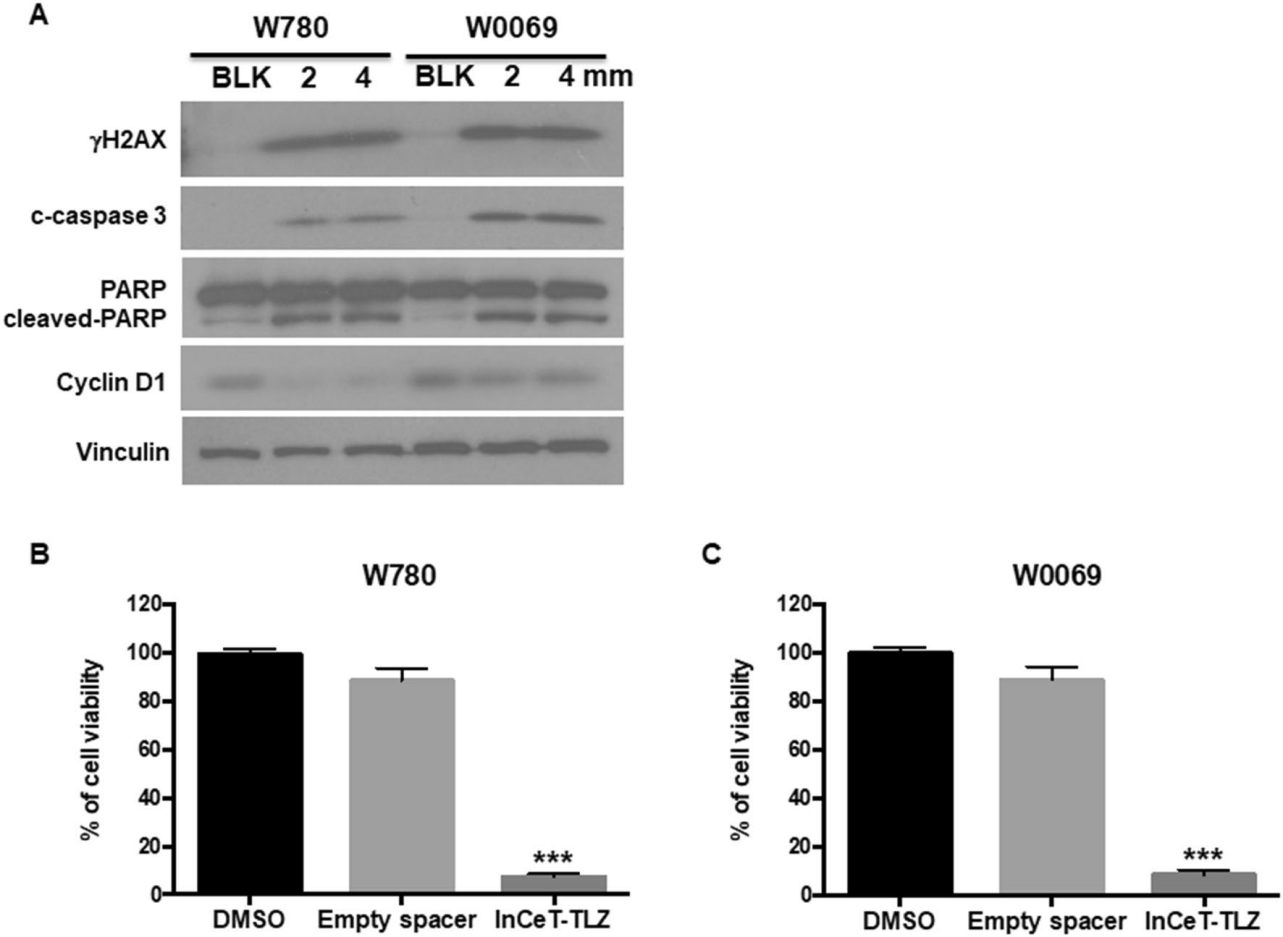
InCeT-TLZ induces DNA damage and cell death in BRCA1-deficient tumor cells. (**A**) BRCA-deficient cancer cells (W780 and W0069) were treated with 2 or 4 mm InCeT-TLZ for 48 h. Biomarkers of DNA damage (γH2AX), cell death (cleaved (c)-caspase 3 and cleaved-PARP) and cell cycle arrest (cyclin D1) were detected by western blotting. W780 (**B**) and W0069 (**C**) were treated with a 1 mm empty spacer or InCeT-TLZ for 5 days. Cell viability was detected using the MTT assay. Results were normalized to the DMSO control. Data was shown as mean ± SD. ***p < 0.001 vs. DMSO control.

To validate the biological effects of InCeT-TLZ in vitro, BRCA1-deficient cancer cells (W780 and W0069) were treated with InCeT-TLZ at the indicated concentrations for 48 h. Biomarkers of DNA damage (γH2AX) and cell death (cleaved-caspase 3 and cleaved-PARP) were detected. Both W780 and W0069 cells were derived from tumors that developed in BRCA1-deficient mice. Although W780 and W0069 share a similar genotype, W780 cells represent an adenocarcinoma phenotype and W0069 cells represent a fibroadenoma phenotype, which is representative of the heterogeneous histopathology found in these tumors in vivo^25^. Talazoparib increased the expression of γH2AX, cleaved-caspase 3, and cleaved-PARP within 48 h^24^. Similarly, treatment with InCeT-TLZ increased the expression of γH2AX, cleaved-caspase 3 and cleaved-PARP in both cell lines (Fig. 2A). In addition, InCeT-TLZ decreased the expression of cyclin D1 (Fig. 2A, Supplementary Fig. 1), suggesting cell cycle arrest, which has been reported for TLZ^24^.

To test the chemical stability and efficacy of drug in the implant, BRCA1-mutated breast tumor cells were incubated with the implants (1 mm) for 5 days. Cell viability was detected using a MTT assay. An empty spacer (1 mm) control was included in the assay in order to analyze the effects of the polymer matrix of the implant. After 5 days of incubation, the cell viability in the InCeT-TLZ implant and empty spacer treated groups were 7.22 ± 0.49% (p < 0.001 vs. DMSO control) and 88.6 ± 2.06%, respectively in W780 cells (Fig. 2B). A similar result was obtained in W0069 cells with 8.75 ± 0.73% (p < 0.001 vs. DMSO control) and 88.9 ± 2.21% cell viability after treatment with the InCeT-TLZ implant and empty spacer, respectively (Fig. 2C). These results confirmed the chemical stability and potency of the drug in the InCeT-TLZ implant. Empty implants did not show any significant toxicity because of the biocompatible nature of PLGA.

### InCeT-TLZ prevents the development of hyperplasia in BRCA1-deficient mice

To evaluate the effects of InCeT-TLZ in vivo, we implanted the InCeT-TLZ spacers into the abdominal mammary glands of Brca1^Co/Co^;MMTV-Cre;p53^+/−^ mice. With conditional knockout of *BRCA1* in their mammary gland, these mice spontaneously develop mammary gland tumors with diverse histopathology at an average age of 24–32 weeks^25^. This genetically engineered mouse model is commonly used to study BRCA1 deficiency and the sensitivity of PARP inhibitors^20,26^. We included three different treatment groups, each with three different starting times for treatment. Treatments were started when the mice were 12, 16, or 20 weeks of age (N = 11–13 mice/group/time point), before any tumors had developed. Age-matched female Brca1^Co/Co^;MMTV-Cre;p53^+/−^ mice were randomized into three treatment groups. The mice in the InCeT-TLZ treatment group were implanted with a TLZ spacer into the left abdominal mammary gland (50 μg drug load, 2 mm in length) using 18G brachytherapy needles. The second group was implanted with an empty spacer (2 mm) as the blank control group. To compare to the oral delivery platform, an oral TLZ group was included (FreeTLZ) as the third treatment group. 50 μg free TLZ was divided into 13 injections and given by gavage three times a week (M, W, F) for 4 weeks (~ 3.84 μg per injection). All the mice were sacrificed 4 weeks after treatment and the left abdominal mammary glands were collected for analysis. Although cohorts of age-matched mice were started on treatment at different ages, the changes across the three treatment groups were consistent. Thus, the three different time points were pooled for data analysis.

**Figure 3:**
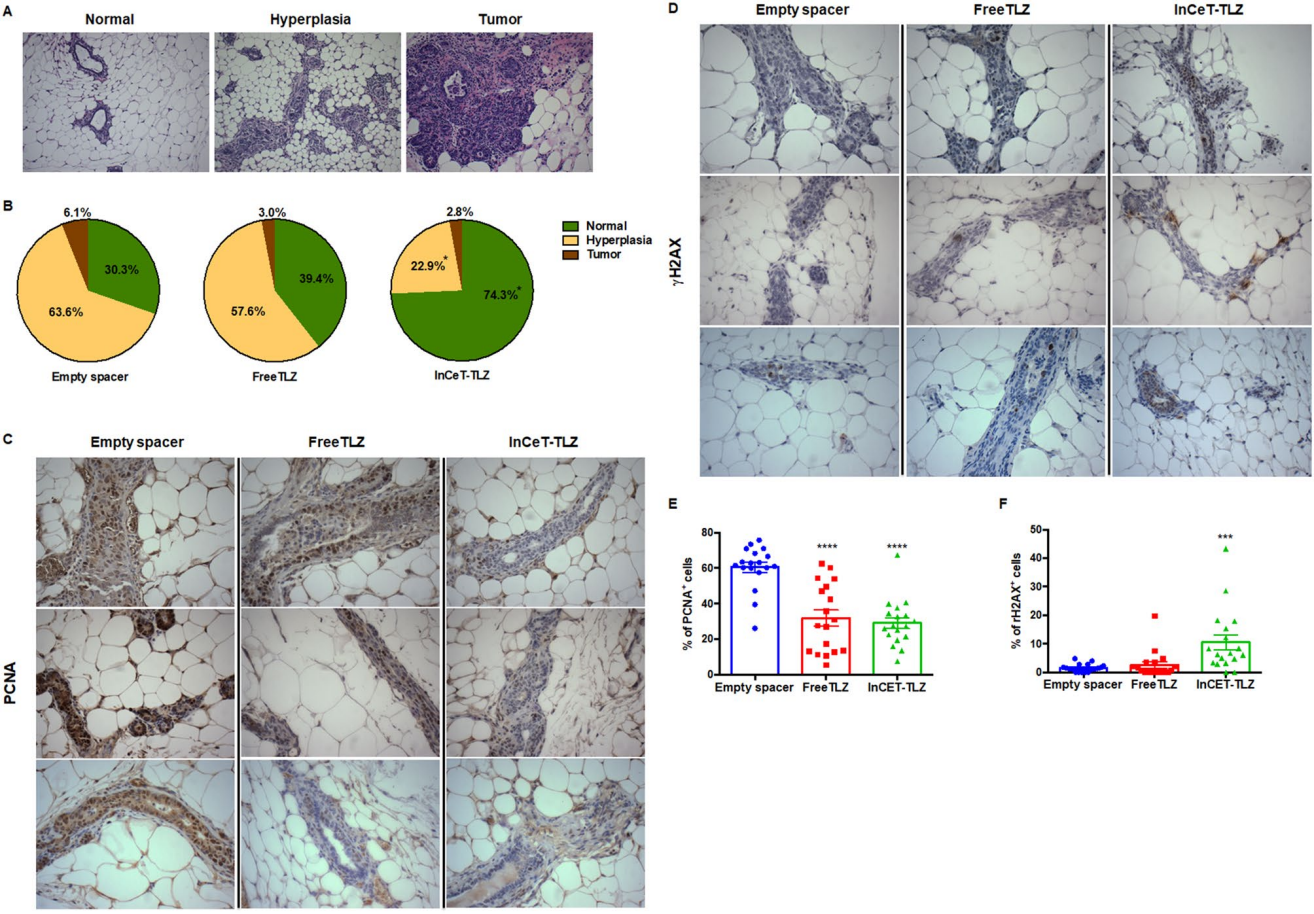
InCeT-TLZ prevents the development of hyperplasia in BRCA1-deficient mice. 2 mm blank implants or 2 mm InCeT-TLZ spacers were injected into the left abdominal mammary gland of Brca1^Co/Co^;MMTV-Cre;p53^+/−^ mice. The same amount of drug (50 μg divided into 13 doses) was given to a third group of mice by gavage as an oral delivery control group (FreeTLZ). All the mice were harvested 4 weeks after treatment, and their mammary glands were collected for analysis. (**A**) Representative H&E staining showing a normal mammary gland, hyperplasia and a mammary gland tumor. ×100 magnification. (**B**) Distribution of histopathology in each treatment group. N = 33–35 mice/group. *p < 0.05 vs. empty spacer group and FreeTLZ. (**C**) Representative pictures of PCNA expression or of γH2AX expression (**D**) in the mammary gland by IHC staining. ×400 magnification. Expression of PCNA (**E**) or γH2AX (**F**) in the mammary gland was quantified using ImageJ. N = 18 mice/group. ****p < 0.0001 vs. empty spacer; ***p < 0.001 vs. empty spacer.

By the chosen end points (16, 20, 24 weeks of age), the majority of the mice had not developed palpable tumors. As expected, only mice from the 24-week-old cohort had developed tumors: two mice in the empty spacer group and one mouse each from the InCeT-TLZ and FreeTLZ groups. Despite the lack of malignant tumors, many of the mice displayed pre-malignant lesions, or hyperplasias, in the mammary gland (Fig. 3A). In the empty spacer group, 63.6% of mice (21 out of 33) developed hyperplasia (Fig. 3B). With the free TLZ treatment, this percentage was slightly lower, but not statistically different, than the empty spacer control group. In the FreeTLZ group, 57.6% of mice (19 out of 33) developed hyperplasia (Fig. 3B). In contrast, treatment with InCeT-TLZ significantly (p < 0.05) decreased the percentage of mice that developed hyperplasia, as only 8 out of 35 mice (22.9%) in this group developed premalignant lesions (Fig. 3B).

DNA damage and cell proliferation have often been used as biomarkers for PARP inhibitors in vivo^24^. By blocking DNA repair pathways, DNA damage and growth arrest are induced in the cells. We stained for the expression of PCNA and γH2AX in mammary gland tissues to evaluate cell proliferation and DNA damage, respectively. In the empty spacer control group, epithelial cells of the ducts were highly proliferative as shown by the dark brown PCNA+ staining (Fig. 3C). DNA damage at this basal level is very rare as shown by the low expression of γH2AX in the mammary gland (Fig. 3D). Both InCeT-TLZ and FreeTLZ treatment arrested cell growth and significantly (p < 0.05) decreased the expression of PCNA in the mammary glands (Fig. 3E). InCeT-TLZ, but not FreeTLZ, induced more DNA damage and significantly (p < 0.05) increased the percentage of cells that are γH2AX positive compared to the control group (Fig. 3F).

### InCeT-TLZ is better tolerated compared to oral TLZ treatment

**Figure. 4:**
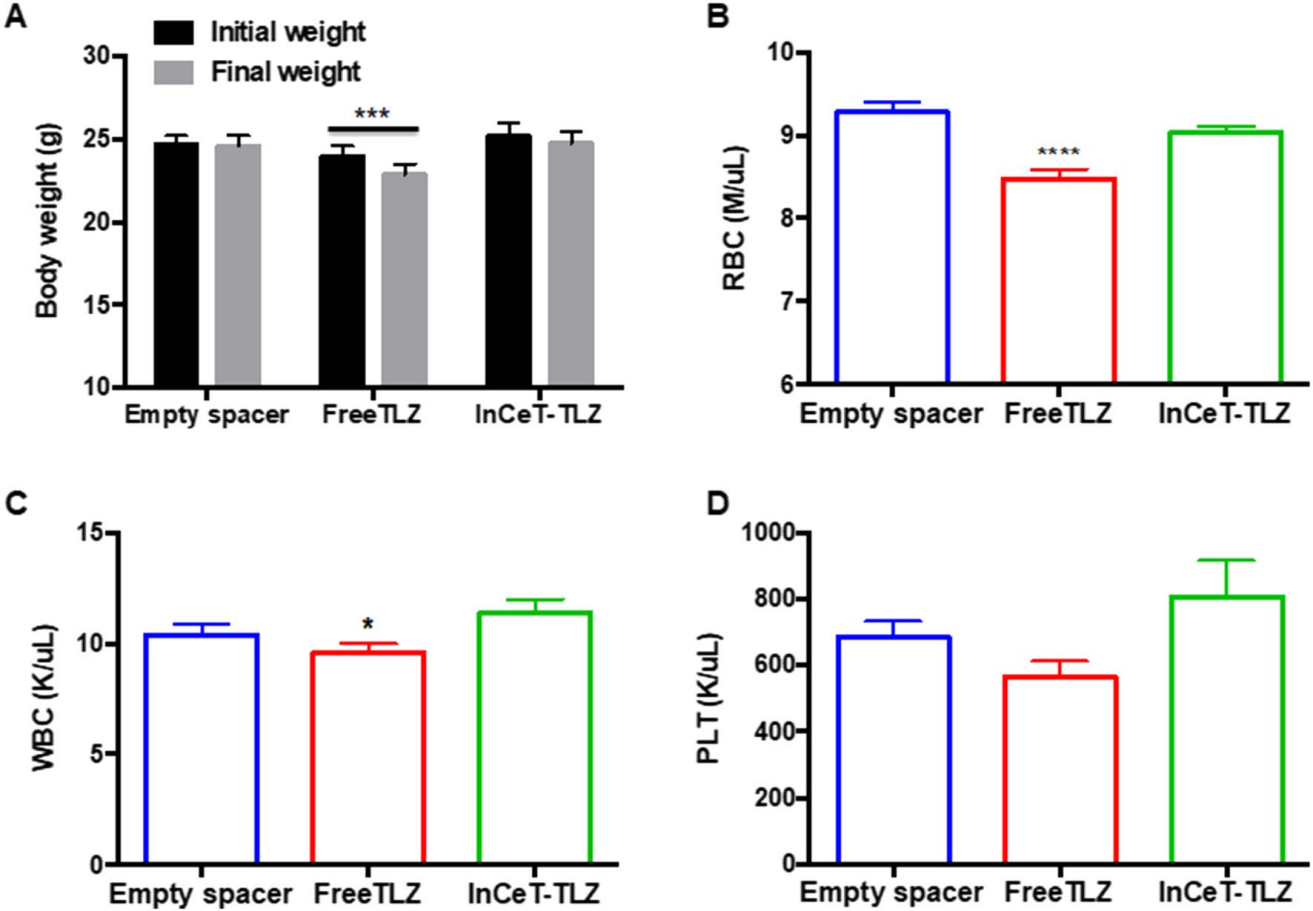
InCeT-TLZ is better tolerated than oral TLZ treatment. (**A**) Body weight of mice before initiation and at the end of treatment. N = 33–35 mice/group. ***p < 0.001 vs. initial weight. (**B**) Red blood cell count (**B**), white blood cell count (**C**) or platelet count (**D**) after 4 weeks of treatment; N = 33 mice/group. ****p < 0.0001 vs. empty spacer; *p < 0.05 vs. InCeT-TLZ.

In addition to efficacy, safety is extremely important for chemopreventive agents. In the clinic, common side effects of talazoparib include fatigue, anemia, thrombocytopenia, neutropenia, alopecia and decreased appetite^27^. To evaluate the toxicity profile of InCeT-TLZ vs. FreeTLZ, we closely monitored body weight as a gross indicator of toxicity. All the mice were weighed twice a week during the 4-week treatment. Treatment with FreeTLZ significantly (p < 0.05) decreased body weight (Fig. 4A). In contrast, no changes in initial vs. final weights were observed in either the empty spacer control group or the InCeT-TLZ treatment group.

Because anemia, neutropenia and thrombocytopenia are known side effects of talazoparib, we performed a complete blood count analysis when the mice were harvested. Indeed, FreeTLZ treatment significantly (p < 0.05) decreased the red blood cell count compared to the control group (Fig. 4B). In contrast, there was no significant change in the red blood cell count between the control group and the InCeT-TLZ treatment group. The number of white blood cells was significantly lower in the FreeTLZ treated mice compared to the InCeT-TLZ group (Fig. 4C). There was also a trend toward a lower platelet count with the FreeTLZ treatment compared to the control group (p = 0.09) that was not observed in the InCeT-TLZ group (Fig. 4D).

## Discussion

In this study, we developed a localized delivery platform for the PARP inhibitor talazoparib as a novel strategy for chemoprevention. We fabricated talazoparib into a spacer implant which can be inserted into the target tissue as a sustained drug release depot. InCeT-TLZ was stable at room temperature and released TLZ in a linear manner over 30 days. Drug loading was consistent across batches (25 μg/mm), and the dose can be adjusted by changing the length of the spacer. InCeT-TLZ did not alter the biological effects of TLZ as it induced DNA damage and cell death in BRCA1-deficient tumor cells. Moreover, InCeT-TLZ, at a low dose, significantly decreased the development of hyperplasia in BRCA-deficient mice and was more efficacious in inducing DNA damage, which is a biomarker for efficacy of PARP inhibitors, compared to empty spacer or oral FreeTLZ treatment. This localized delivery of TLZ was also well tolerated, as no significant weight loss or anemia was observed as in the systemic exposure group. Our results demonstrate that these TLZ spacer implants are effective chemopreventive agents with minimal toxicity in BRCA1-deficient mice. We anticipate the same potential benefits with this approach could be optimized for use in patients with BRCA deficiency, although translation into human patients will require optimization of the number of implants needed based on the diffusion of drug out of the spacer.

Besides the well-defined high-risk patients with known germline *BRCA* mutations, many other people could potentially benefit from this chemopreventive strategy. PARP inhibitors are proposed to selectively target cells that undergo malignant transformation and acquire defects in DNA repair pathways. In addition to mutations in *BRCA1/2* genes, other genetic alterations could also produce HR deficiency and make cells vulnerable to synthetic lethality^28^. This includes genetic alterations of *RAD51, ATR, CHK1/2, ATM, FANCD2* and *FANCA*, which are all key players within the HR pathway^29^. It has already been shown that PARP inhibitors are effective in treating *BRCA1/2* wild-type tumors^30^. Therefore, InCeT-TLZ could potentially impact more women beyond populations with *BRCA* mutations. Additional studies should test the InCeT-TLZ platform for preventing other subtypes of breast cancer.

Chemoprevention has been an underused approach to reduce cancer risk and mortality. The selective estrogen receptor modulators (SERMs) tamoxifen and raloxifene remain the only approved drugs for breast cancer prevention over the last 20 years. Unfortunately, the ineffectiveness of SERMs in preventing BRCA-deficient breast cancer leaves bilateral prophylactic mastectomy (BPM) or “watchful waiting” the only options for BRCA carriers. Those not interested in BPM should be offered additional preventive therapies besides surveillance with annual mammograms or magnetic resonance imaging^7^.

A number of compounds, including PARP inhibitors (olaparib and veliparib)^20,26^, have shown efficacy in preventing or delaying breast cancer development in mouse models. Our study indicates TLZ, a more potent PARP inhibitor compared to olaparib and veliparib, could also serve as a potential chemopreventive agent for breast cancer. The synthetic triterpenoid CDDO-methyl ester has been tested in the BRCA-deficient mouse model and significantly delayed tumor development by an average of 5.2 weeks^23^. The synthetic retinoid fenretinide has shown promising results in clinical trials for breast cancer chemoprevention^31^. The bromodomain inhibitor I-BET 762 delays tumor onset in a mouse model of ER^-^ breast cancer^32^. The histone deacetylase inhibitor vorinostat, alone or in combination with synthetic triterpenoids, also reduces tumorigenesis in the PyMT breast cancer mouse model^33^. Drugs that are approved and widely used for diseases other than cancer can also be explored for chemopreventive potential. Successful chemoprevention agents require comprehensive evaluation to balance between risk and benefits.

A localized delivery platform is one way to lower risk and enhance efficacy. Sustained and local delivery of drugs becomes an option with the use of biodegradable materials. PLGA is a FDA-approved polymer with great biocompatibility and biodegradability. It has been broadly used to develop controlled delivery systems for drugs from small molecules, macromolecules or proteins^34^. Here, we fabricated TLZ into PLGA spacers, which can be easily adapted using existing brachytherapy procedures. As a localized treatment, InCeT-TLZ could overcome some of the limitations of conventional oral delivery of PARP inhibitors, such as limited bioavailability, poor tissue accumulation, and early drug metabolism. By avoiding a first pass through the systemic circulation, local delivery also reduces the risk of systemic toxicities. Unlike other strategies, such as intermittent dosing, this implantable formulation bypasses the concerns of noncompliance in patients and further improves the therapeutic effects. The differences of effects between local delivery and intermittent therapy needs to be compared directly in future studies. Our proof-of-concept study provided a template that can be easily used to formulate other drugs for many other applications.

Biomarker studies may serve as a model to screen new agents efficiently. Biomarkers are not only powerful tools for disease diagnosis and personalized medication, they are also playing increasing roles in drug discovery and development^35^. By knowing the mechanism of action of a drug, predictive biomarkers can be used to evaluate drug efficacy and toxicity at early stages. Biomarkers are often evaluated as surrogate endpoints in chemopreventive clinical trials. For instance, histological modulation of intraepithelial neoplasias has been the primary phenotypic surrogate end point in the National Cancer Institute chemoprevention program^36^. Biomarkers of proliferation, differentiation, chromosomal damage, cell growth regulatory molecules, and biochemical activities are other potential surrogate end points^36^.

In our study, we have confirmed the biological changes induced by the PARP inhibitor talazoparib. Induction of γH2AX, in particular, has been commonly used as an indicator of drug response^37^. The decreased incidence of hyperplasia also suggests a promising chemopreventive effect of InCeT-TLZ. Although systemic administration of PARP inhibitors has shown efficacy in previous publications^20^, FreeTLZ failed to reduce the percentage of hyperplasia cases in our study. The main reason for the difference is that we used a much lower dose of talazoparib compared to previous studies. In the systemic administration (FreeTLZ) group, a total of 50 μg TLZ was divided into 13 injections and given 3 times a week over 4 weeks, which translates to only 3.85 μg per injection and approximately 0.13 mg/kg body weight. In previous prevention studies, up to 200 mg/kg diet olaparib (roughly 50 mg/kg body weight) was used. With a lower dose, lower efficacy was expected. Indeed, FreeTLZ significantly decreased the percentage of proliferating cells, which is predictive of delayed tumor development for prevention in this model. In future studies, we will directly evaluate the efficacy of InCeT-TLZ for preventing tumor development. Other drug candidates for chemoprevention and possible drug combinations will be explored using this localized delivery platform.

## Supplementary Information

Supplementary Information.

## Abbreviations

ATM: Ataxia telangiesctasia mutated gene
ATR: Ataxia telangiectasia and Rad3 related
BPM: Bilateral prophylactic mastectomy
BRCA: Breast cancer-associated gene
CHK1/2: Checkpoint kinase 1/2
FANCA: FA Complementation Group A
FANCD2: FA Complementation Group D2
HR: Homologous recombination
InCeT-TLZ: Talazoparib (TLZ) spacer implants
SERMs: Selective estrogen receptor modulators
PARP: Poly (ADP-ribose) polymerase
PCNA: Proliferating cell nuclear antigen
PLGA: Poly (lactic-co-glycolic) acid
