# Supplementary material for: Sustained, local delivery of the PARP inhibitor talazoparib prevents the development of mammary gland hyperplasia in Brca1-deficient mice
Source: Sci Rep. 2021 Jan 13;11:1234. doi: 10.1038/s41598-020-79663-7 (PMC7806744; doi:10.1038/s41598-020-79663-7)
Supplement: Supplementary file 1 — Supplementary Information. [file 41598_2020_79663_MOESM1_ESM.pdf]

Sustained, local delivery of the PARP inhibitor talazoparib prevents the development of mammary gland hyperplasia in *Brca1*-deficient mice

Di Zhang[1*], Bijay Singh[2,3*], Jessica Moerland[1], Owen Mitchell[1], Lizbeth Lockwood[1], Sarah Carapellucci[1], Srinivas Sridhar[2,3], Karen T. Liby[1]

Authors' affiliations: [1]Michigan State University, East Lansing, MI; [2]Theranano LLC, Newton, MA; [3]Northeastern University, Boston, MA.

[*] These authors contributed equally to this work.

Exposure of γH2AX and Vinculin

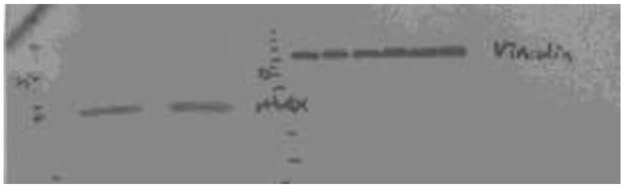

Longer exposure of γH2AX

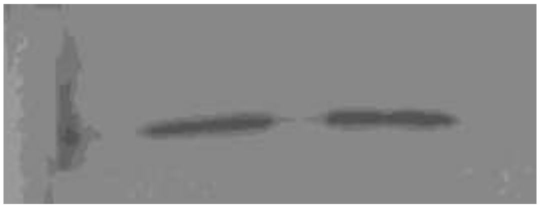

Shorter exposure of Vinculin

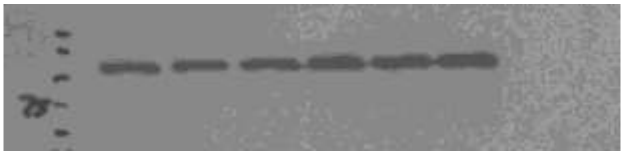

Exposure of c-caspase 3

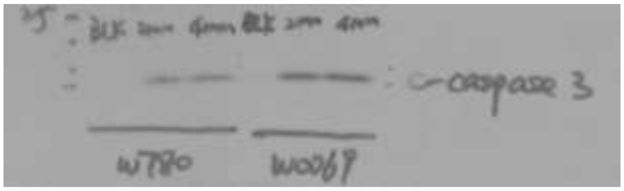

Exposure of PARP and cleaved-PARP

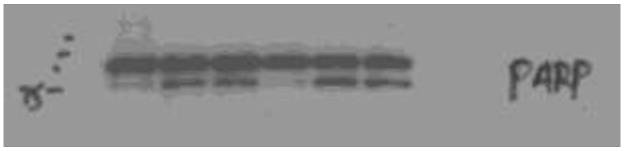

Exposure of Cyclin D1

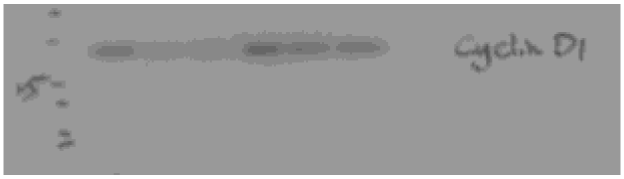

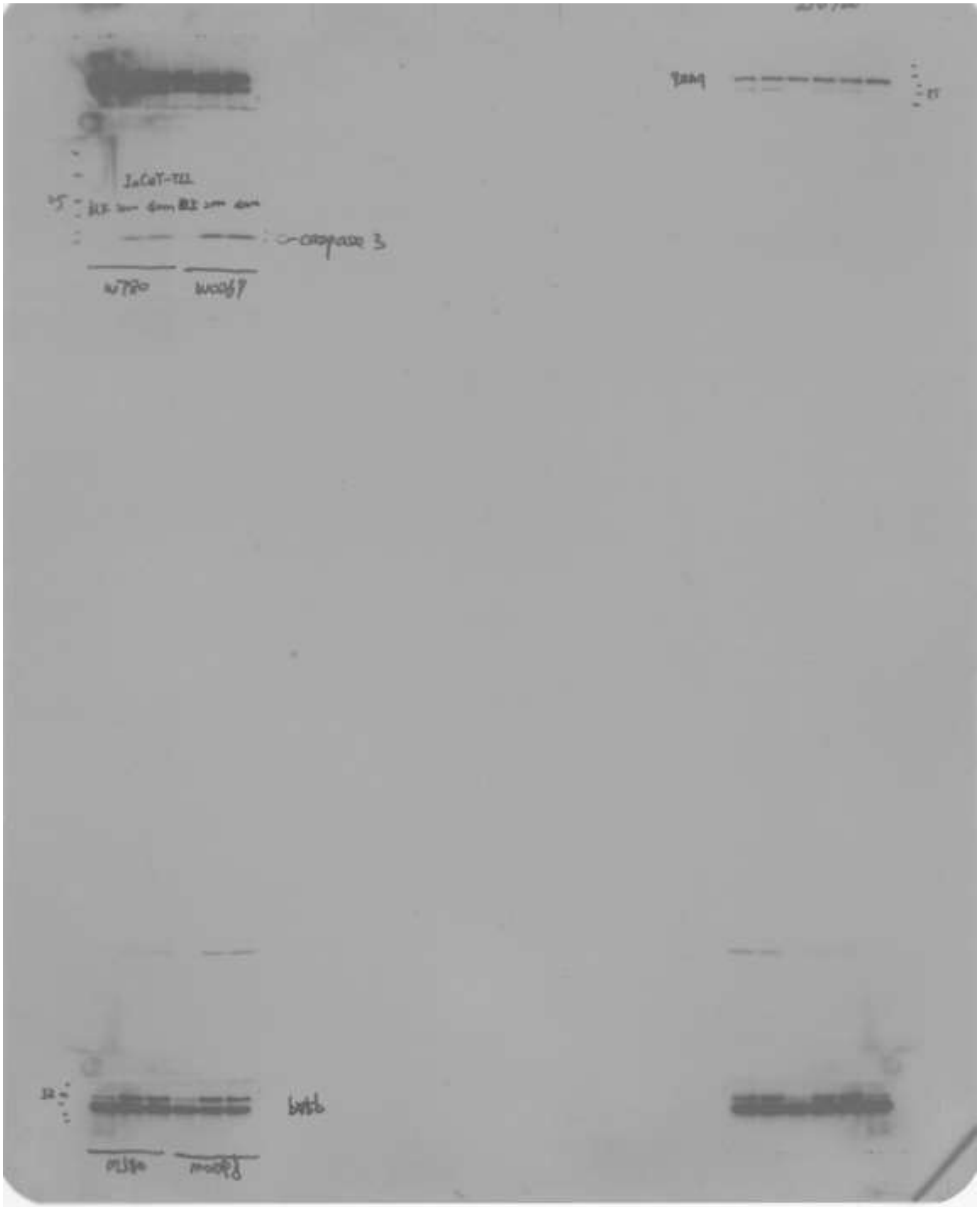
caspase 3

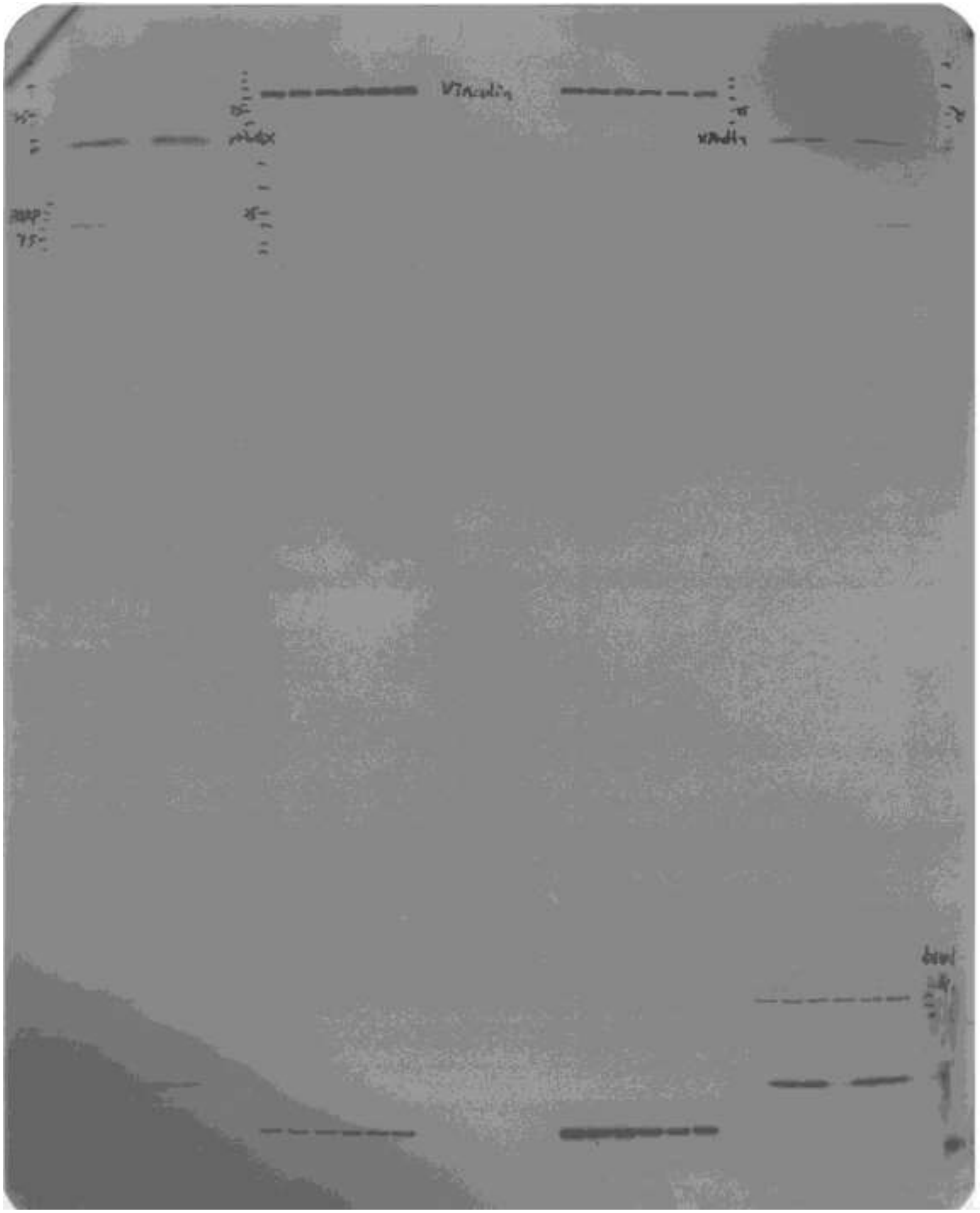

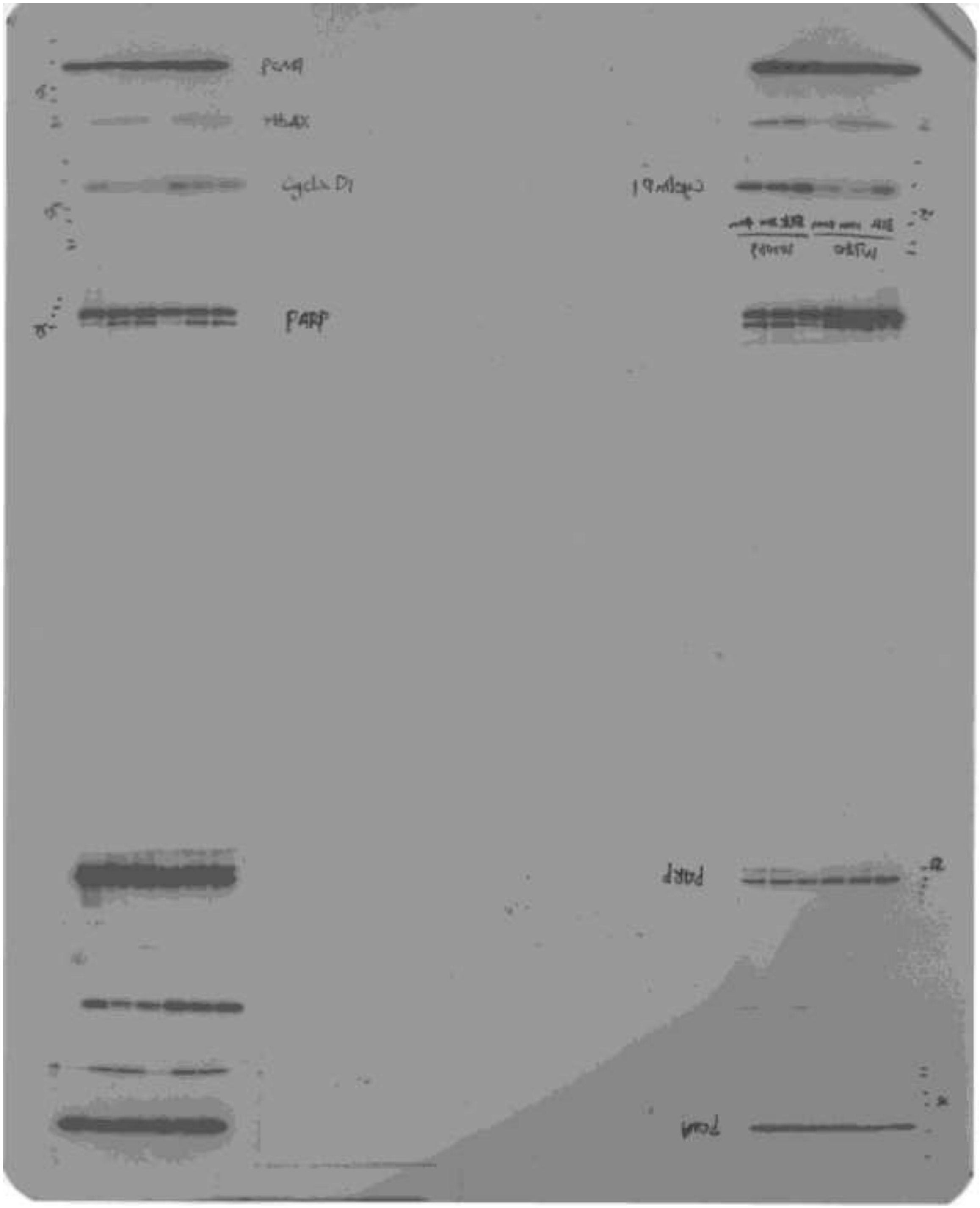

Suppl. Fig.1: Immunoblots used in Fig.2A.
